# Experimental Butchering of a Chimpanzee Carcass for Archaeological Purposes

**DOI:** 10.1371/journal.pone.0121208

**Published:** 2015-03-20

**Authors:** Palmira Saladié, Isabel Cáceres, Rosa Huguet, Antonio Rodríguez-Hidalgo, Borís Santander, Andreu Ollé, Mª Joana Gabucio, Patricia Martín, Juan Marín

**Affiliations:** 1 IPHES, Institut Català de Paleoecologia Humana i Evolució Social, Tarragona, Spain; 2 Área de Prehistoria, Universitat Rovira i Virgili (URV), Tarragona, Spain; 3 GQP-CG, Grupo Quaternário e Pré-História do Centro de Geociências (uI and D 73—FCT), Tomar, Portugal; 4 Unit associated to Consejo Superior de Investigaciones Ciéntíficas (CSIC), Departamento de Paleobiologia, Museo de Ciencias Naturales (MNCN), Madrid, Spain; 5 Equipo Primeros Pobladores de Extremadura, Cáceres, Spain; University of Oxford, UNITED KINGDOM

## Abstract

Two archaeological assemblages from the Sierra de Atapuerca sites show evidence of anthropogenic cannibalism. These are the late Early Pleistocene level TD6-2 at Gran Dolina, and the Bronze Age level MIR4 in the Mirador Cave. Despite the chronological distance between these two assemblages, they share the common feature that the human remains exhibit a high frequency of anthropogenic modifications (cut marks, percussion pits and notches and peeling). This frequency could denote special treatment of bodies, or else be the normal result of the butchering process. In order to test these possibilities, we subjected a chimpanzee carcass to a butchering process. The processing was intensive and intended to simulate preparation for consumption. In doing this, we used several simple flakes made from quartzite and chert from quarries in the Sierra de Atapuerca. The skull, long bones, metapodials and phalanges were also fractured in order to remove the brain and bone marrow. As a result, about 40% of the remains showed some kind of human modification. The frequency, distribution and characteristics of these modifications are very similar to those documented on the remains of *Homo antecessor* from TD6-2. In case of the MIR4 assemblage, the results are similar except in the treatment of skulls. Our results indicate that high frequencies of anthropogenic modifications are common after an intensive butchering process intended to prepare a hominin body for consumption in different contexts (both where there was possible ritual behavior and where this was not the case and the modifications are not the result of special treatment).

## Introduction

Among the archaeological deposits in the Sierra de Atapuerca (Burgos, Spain), two assemblages show evidence of cannibalism. These are the late Early Pleistocene level TD6–2 of Gran Dolina and the Bronze Age level MIR4 in Mirador Cave. Remains of hominins/humans (hereafter referred to as human remains) were recovered with obvious evidence of butchering and consumption [1–6]. All of these studies concluded that human bodies were processed in the same way as animal carcasses. However, in both assemblages, a higher number of cut marks were documented on the human remains than on other animal remains [4,5,7]. This tendency has also been observed in other cannibalized assemblages, such as Moula Guercy (France) [8,9] and Brillenhöhle (southwestern Germany) [10], in which the percentage of human bones with cut marks ranges between 40 and 60%.

The cut marks, along with the anthropogenic bone breakage, can provide a remarkable amount of paleo-economic information about prehistoric human groups. Since the 1980s, a great deal of research has focused on the macroscopic and microscopic morphology of cut marks [11–14] and their distribution on the skeleton and on different parts of the bones in order to infer information such as the timing of access that Pleistocene hominins had to carcasses [15–18], the handedness [19] and, more recently, Stiner et al.[20]suggested that through the orientation of cut marks may determine if one or several individuals were involved in the butchering. However, Egeland [21] indicates that the orientation diversity is related to the skill and experience of the butchers.

In this line of research, several proposals have been put forth as to the different variables that may influence the frequency of cut marks. Binford [22] suggested that when the burden of extracting the tissue is lower and, therefore, more scraps of meat are attached to the bone, fewer marks should be expected. The frequency of cut marks would therefore be related, according to these observations, to the intensity of the butchering process. Milo [23] supported a similar argument, although she added, as another variable, the difficulty in processing of carcasses. None of these assessments, however, was supported by actualistic data to substantiate these hypotheses.

Egeland [21] used an experimental approach to test the butchering process intensity and its relationship to the frequency of cut marks with quantitative data. For this purpose, he proposed studying the relationship between two simple measurements: the number of arm movements made during defleshing activities, and resulting number of individual striae. Experimental data obtained by Egeland [21] indicates that the relationship between increased frequencies of cut marks and more intensive processing is not consistent. Previously, Lyman [24] had suggested that the variations in cut marks frequency were related to the size of the animals butchered, with more cut marks on larger specimens. He later, however, needed to expand his explanatory models to account for the diversity of frequencies of butchered bones intra and inter-assemblages [25]. And he finally claimed that the interpretations of cut mark frequencies may require unique contextual data in each specific assemblage [26].

Dewbury and Russell [27] concluded that the raw material of the stone tools used is another variable that can affect the frequency of marks. Although their results were not statistically significant, they claim that this variable should be taken into consideration. In addition, Padilla [28]showed that, in butchery experiments, the frequencies of cut marks vary depending on the experience and skill of the butchers.

Finally, Domínguez-Rodrigo and Yravedra [29] proposed three major variables as significant factors: (1) the size of carcass and type of tool (lithic or metal); (2) bone breakage and (3) ravaging by carnivores and the consequent damages to the skeletal profiles in the original samples. According to Domínguez-Rodrigo and Yravedra [29], skeletal profiles ‒ regardless of their origin and cause ‒ may influence the proportion of bones bearing butchery marks.

In this context, our goal is to create an experimental framework that would allow us to describe the butchery activities performed on the cannibalized human corpses recovered from level TD6–2 of Gran Dolina and level MIR-4 of the Mirador Cave. We have developed this research starting from an uniformitarian assumption. The specific objective of this experimental work is to provide actualistic observations for the interpretation of the frequency of cut marks on human remains. It is difficult to perform these experiments with human bodies. For this reason when the opportunity arose we decided that the processing of the carcass of a chimp could be helpful to our goal. We have created this actualistic framework through carrying out the butchering process (bone breakage included) on the carcass of a chimpanzee. We must point out that although there are similarities between chimpanzees and humans, we are also aware that there are considerable differences. The most important of these differences is that humans are bipedal while chimpanzees are terrestrial quadrupeds that support their forelimbs on their knuckles and not on the palms of their hands or paws like other quadrupeds. However, they can stand up and walk on their legs (bipedally) when circumstances require freeing up their hands for use. This characteristic makes the anatomy of these primates closer to hominins than that of any fully quadruped mammals, despite the clear differences in the proportions of the segments of the body.

### Archaeological context

The Atapuerca complex (Burgos, Spain) contains several archaeological and palaeontological sites dating from the Early Pleistocene to the Holocene [30–34] Evidence of cannibalism has been recorded in two assemblages from La Sierra de Atapuerca.

#### TD6–2 assemblage

The oldest evidence of the practice of cannibalism among hominins was found in level TD6–2 at the Gran Dolina site (Sierra de Atapuerca, Burgos, Spain). Several stratigraphic studies have established that level TD6 dates to the late Early Pleistocene [35–39]. *H*.*antecessor* remains were recovered [40], along with a large number of faunal specimens and over 600 lithic artifacts. The lithic assemblage is attributed to Mode 1 technology [32,41].

The hominin remains exhibit several anthropogenic modifications on their surfaces which indicate that they had been processed and consumed by other hominins [3,5]

After a new review of the TD6–2 assemblage, *H*. *antecessor* sample was found to contain 181 remains (155 bones and 26 teeth) from 11 individuals (MNI). Cut marks are present on all types of bones (Table 1). Additionally, there is abundant evidence of anthropogenic bone breakage (percussion pits/notches or peeling). In total, 44.5% of the bones of *H*. *antecessor* exhibit some type of anthropogenic modification. Damage resulting from non-human carnivore activity has not been documented on the *H*. *antecessor* remains [42].

**Table 1:** Anthropogenic modifications on bone of principal taxa from TD6–2 (*Homo antecessor* and Cervidae 2–3 sized) and MIR4 (*Homo sapiens* and Ovicaprini).

	TD6–2						MIR4					
	*Homo antecessor*			Size 2–3 Cervidae			*Homo sapiens*			Ovicaprini		
	NISP	Cut marks	Anthrop. breakage*	NISP	Cut marks	Anthrop. breakage*	NISP	Cut marks	Anthrop. breakage*	NISP	Cut marks	Anthrop. breakage*
**Skull**	25	7 (28%)	7 (28%)	3	0 (0%)	0 (0%)	42	26 (61.9%)	12 (28.6%)	5	0 (0%)	0 (0%)
**Mandible**	5	2 (40%)	1(20%)	11	2 (18.2%)	0 (0%)	7	7 (100%)	5 (71.4%)	6	2 (33.3%)	1 (16.6%)
**Vertebrae**	20	5 (25%)	6 (30%)	4	1 (25%)	1 (25%)	15	5 (33.3%)	8 (53.3%)	9	3 (33.3%)	0 (0%)
**Clavicle**	3	3 (100%)	0 (0%)	-	-	-	1	1 (100%)	1 (100%)	-	-	-
**Ribs**	43	15 (34.9%)	11(25.6%)	4	1 (25%)	0 (0%)	18	6 (33.3%)	8 (44.4%)	6	2 (33.3%)	0 (0%)
**Coxa**	2	1 (50%)	0 (0%)	0	0 (0%)	0 (0%)	2	0 (0%)	2 (50%)	4	0 (0%)	2 (50%)
**Scapula**	3	1 (33.3%)	0 (0%)	8	1 (12.5%)	2 (25%)	8	4 (50%)	4 (50%)	4	0 (0%)	0 (0%)
**Humerus**	3	1 (33.3%)	0 (0%)	39	18 (46.1%)	5 (12.8%)	6	4 (66.6%)	4 (66.6%)	8	5 (62.5)	1 (12.5%)
**Radi**	2	2 (100%)	2 (100%)	32	10 (31.2%)	4 (12.5%)	1	0 (0%)	0 (0%)	6	2 (33.3%)	0 (0%)
**Ulna**	2	2 (100%)	2 (100%)	10	2(20%)	2 (20%)	4	3 (75%)	1 (25%)	1	0 (0%)	0 (0%)
**Metacarpal**	2	1 (50%)	1 (50%)	32	3 (9.4%)	2 (6.2%)	4	0 (0%)	0 (0%)	5	0 (0%)	0 (0%)
**Femur**	4	4 (100%)	2 (50%)	44	13 (29.5%)	4 (9%)	6	1 (16.7%)	0 (0%)	6	1 (16.6%)	0 (0%)
**Tibia**	2	2 (100%)	1 (5%)	30	11 (36.6%)	4 (13.3%)	13	7 (53.8%)	8 (61.5%)	7	1 (14.3%)	1 (14.3%)
**Fibula**	2	1 (50%)	0 (0%)	0	0 (0%)	0 (0%)	5	2 (40%)	4 (20%)	0	0 (0%)	0 (0%)
**Metatarsal**	5	3 (60%)	3 (60%)	30	3 (10%)	2 (6.6%)	4	1 (25%)	0 (0%)	2	0 (0%)	0 (0%)
**Carpal/tarsal/ Sesamoide**	6	0 (0%)	0 (0%)	21	1 (4.6%)	0 (0%)	4	0 (0%)	0 (0%)	5	1 (20%)	0 (0%)
**Phalanges**	24	4 (16.6%)	2 (8.3%)	16	2 (12.5%)	1 (6.2%)	8	0 (0%)	0 (0%)	4	0 (0%)	0 (0%)
**Total**	155	50 (32.2%)	38 (24.5%)	293	69 23.5%	28 (9.5%)	148	67 (45.3%)	57 (38.5%)	79	17 (21.5%)	10 (12.65)
**Total anthrop. modifications**	69 (44.5%)			87 (29.7%)			89 (60.1%)			23 (29.1%)		

Along with hominin specimens, the most abundant taxa recovered were medium-sized ungulates, represented mainly by deer. Of the Cervidae remains, 29.7% exhibit anthropogenic modifications (Table 1) and 10.6% show carnivore tooth marks (Saladié et al., 2012).

#### MIR4 assemblage

Mirador Cave is located on the southern slope of the Sierra de Atapuerca. In level MIR 4, a 6 m^2^test pit revealed an intentional accumulation of human remains, from which both bones and teeth were recovered (MIR4A). All the remains had been deposited in a small (40 x 25 x 10 cm) oval pit [43]. Charcoal and bone dating places the chronology of level MIR4 in the Middle Bronze Age, during which time the cave was used as an animal shelter. The human remains, however, provide calibrated dates between 4400 and 4100 BP. The dates indicate that the human remains date from the Early Bronze Age, while the burial event must have occurred during the Middle or Late Bronze Age[1]

The skeletal remains of six individuals, consisting of a total of 165 specimens (148 bones and 17 isolated teeth) were recovered from MIR4A [1,4]. Taphonomic analysis revealed that the individuals had undergone an intense defleshing process, and most specimens exhibited human tooth marks and evidence of boiling and intentional bone breakage. Skulls displayed a well-defined breakage pattern, with conchoidal scars around the entire perimeter of the skull and percussion marks on the temporal, parietal and occipital bones. These modifications suggest consumption as the main objective of processing the bodies. However, the remains were clearly buried intentionally in a specific way, with the skulls carefully arranged at the bottom of a pit and the remaining fragments on top of them, although the dates of some human bones and charcoal seem to indicate that the remains were buried by people unrelated to the cannibalism event several centuries afterwards. A review of the fauna from this level[4] revealed no faunal remains inside the pit and that some human remains were dispersed across other parts of the surface and mixed with different archaeological material. Also, while the humans had only been boiled, the remains of other fauna showed evidence of being both roasted and boiled. These features, along with the dating, indicate that the remains of ungulates and human remains from the pit do not correspond to the same event and that the human remains are older than the other remains at the site. This may also alter the skeletal profile represented in the pit due to bone selection that occurred during the secondary burial.

Table 1 shows the percentage of human modifications on the *H*. *sapiens* and ovicaprini remains from MIR4A and MIR4. This emphasizes the high degree to which anthropogenic modifications are present, as they were found on over 60% of the hominin assemblage. Ovicaprini remains exhibit anthropogenic modifications on fewer than 30% of the assemblage.

## Materials and Methods

The body of the chimpanzee was made available to the IPHES team by the Fundació Mona primate recovery center (Riudellots de la Selva, Girona, Spain). The Fundació Mona is a nonprofit organization (registration 1404 in the Justice Department of the Generalitat de Catalunya) whose aim is to ensure the welfare of confiscated primates that have been detected in illegal or abuse situations. The foundation has a recovery center close to the city of Girona which is used as a home for rescued primates in order to rehabilitate and protect them. The foundation also has a research unit with an interdisciplinary team of psychologists, biologists, veterinarians and anthropologists. The chimpanzee died under veterinarian care at the Fundació Mona from natural causes at 57 years old. We will not offer images of the process described here out of respect to animal, its caregivers and the center’s generous sponsor.

The chimpanzee had lost most of its dentition and had osteoarthritis in most of its bone articulations, related to its advanced age.

Butchering process was recorded by photographing it and taking notes. In addition, following the advice of Nilssen[44]the entire process was recorded with video camera for later review, in order to expand on the notes and check that they were accurate. The images have also been used in studying the bones, and allowed us to make very reliable links between specific butchering activities and specific modifications.

**Fig 1:**
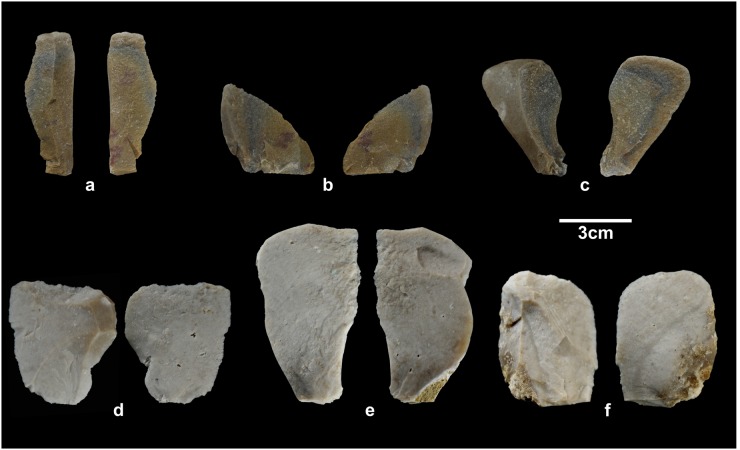
Selection of the quartzite and chert flakes used in the butchery experiment. a) QTAC19; b) QTAC20; c) QTAC21; d) SNC40; e) SNC41 and f) SNC42.

For skinning, defleshing and disarticulation, we used simple chert and quartzite flakes. From the Lower Pleistocene to the Holocene (Fig. 1), chert and quartzite were the main raw materials used for tool production at the Sierra de Atapuerca sites. We chose two specific varieties of stone: Neogene chert from the Late Miocene formations around the Sierra, and quartzite from the Arenas de Utrillas facies [45]. This chert is made up of microcrystalline grains and has moderate to good knapping properties; the quartzite is, in fact, a homogeneous and fine-grained metaquartzite. The specific features of the tools we used are presented in Table 2. Carcass butchering involved three expert butchers, who were assisted by three assistants. One expert removed the skin and butchered the right forelimb, the right hind limb, and trunk. Another butcher processed the left forelimb and the left hind limb. Finally, the third butcher processed the skull and, with an assistant, broke the bones.

**Table 2:** Experimental protocol variables and edge features of the stone tools.

Experimental variables								Tool measures (mm)			Edge features			
Ref	Worked Material	Actions	Motion	α of work	Time	Hand	Exp	L	W	T	α edge	Edge shaping	Prof. Del.	Hor. Del.
**QTAC19**	Skin, subcutaneous tissues	Skinning	Long. Uni/Bid.	75–90°	40 min	R	1	50	19	9	35°	ncfg.	inc	Cx
**QTAC20**	Skin, subcutaneous tissues	Skinning	Long. Uni.	75–90°	15 min	L	2	50	26	7	30°	ncfg.	inc	Cx
**QTAC21**	Meat	Disarticuling	Uni. Long.	75–90°	45 min	R	3	53	29	9	45°	ncfg.	inc	Cx
**SNC40**	Meat	Disarticuling and defleshing	Uni/bid. Long.	75–90°	120 min	R	1	50	46	10	30°	ncfg.	str	Cx(sin)
**SNC41**	Skin / meat	Skinning, disariculing, defleshing	Uni/bid. Trans.	75–90°	35 min	R	1	74	43	21	40°	ncfg.	inc	Cx
**SNC42**	Skin / meat	Skinning, disariculing, defleshing	Uni/bid. Trans.	75–90°	32 min	L	2	57	40	14	40°	ncfg.	inc	cx
**SNC43**	Skin / meat	Skinning, disariculing, defleshing	Uni/bid. Trans.	75–90°	45 min	L	2	36	21	5	35°	ncfg.	inc	cx

Ref. (QTA for quartzite tools and SN for Neogene chert tools); worked material; actions; direction of motion (unidirectional-Uni-, bidirectional-bid-, longitudinal-Long-, transverse-Trans-); angle of work; time: angle edge; edge shaping (shaped-shp- and not sdhaped—nshp-); profile delineation (straight—str-, incurved-inc-, sinuous—sin-); horizontal delineation (convex-cx-, sinuous-sin-, uniangular-1a-).

We have make an ANOVA test to evaluate possible differences in the frequency or the locations of the cut marks produced by the two main butchers involved in processing the chimpanzee carcass. The analytical variables used were: NISP of remains with cut marks on: Clavicles; Os Coxae; Scapulae; Humeri; Radii; Ulnae; Femurs; Tibiae; Fibulae; NISP Total; Maximum number of cut marks on one specimen; Maximum number of cut marks on one element; Total number of cut-marks; NISP of shafts with cut marks; NISP of epiphyses with cut marks.

In the laboratory, bones were boiled to make it easier to remove the meat scraps, tendons and fat. The bones were then dried in the shade. A total of 314 bone specimens were collected. There were also 140 unidentifiable fragments less than 2 cm long that were recorded but not included in the analysis.

The entire surface of every bone was examined, both macroscopically and microscopically (OPTHEC 120HZ), and for each anthropogenic modification (cut marks and bone breakage signals) the location, segment, portion and face were noted.

We recognized three types of cut marks: slicing, scraping and chop marks [46,47]. Slicing marks occur when the tool is applied with force parallel to the long axis of the tool’s edge. Scraping marks occur when the edge is applied perpendicularly to the bone surface, generating multiple unidirectional striations. Finally, chop marks are produced by applying a dynamic force (percussion) using a tool with an edge [48].

The analysis of cut marks took into account the number of individual striae, location on the skeletal element, distribution over the surface (isolated, dispersed all over, clustered) and orientation in relation to the longitudinal axis of the bone (oblique, longitudinal, transverse). Maximum length of the cut marks was measured in millimeters.

We also recorded the presence/absence and location of percussion pits, impact points, adhered flakes on the surface, conchoidal scars, hammerstone/anvil abrasions and peeling on each item studied [49–51]. Peeling was described as general, classical or incipient in keeping with the suggestions of Pickering et al [52] Finally, the experimental results were statistically compared (by correspondence analysis) with for the cut mark results for the hominin and deer (*Cervus/Dama*) remains from level TD6–2 and those for the human and *Ovis/Capra* remainsfrom level MIR4.

### Description of the butchering process

The butchering process of the chimpanzee carcass consisted of making the fullest possible use of the animal by skinning, dismembering, defleshing, disarticulating it and bone breakage to extract the marrow. In this paper, the intensive butchering process is understood to mean removing the maximum amount of soft tissue suitable for consumption. We did not remove the viscera of the chimpanzee because it had undergone a necropsy, so the thoracic cavity had been opened and visceral content had been clinically removed.

#### Skinning

**Fig 2:**
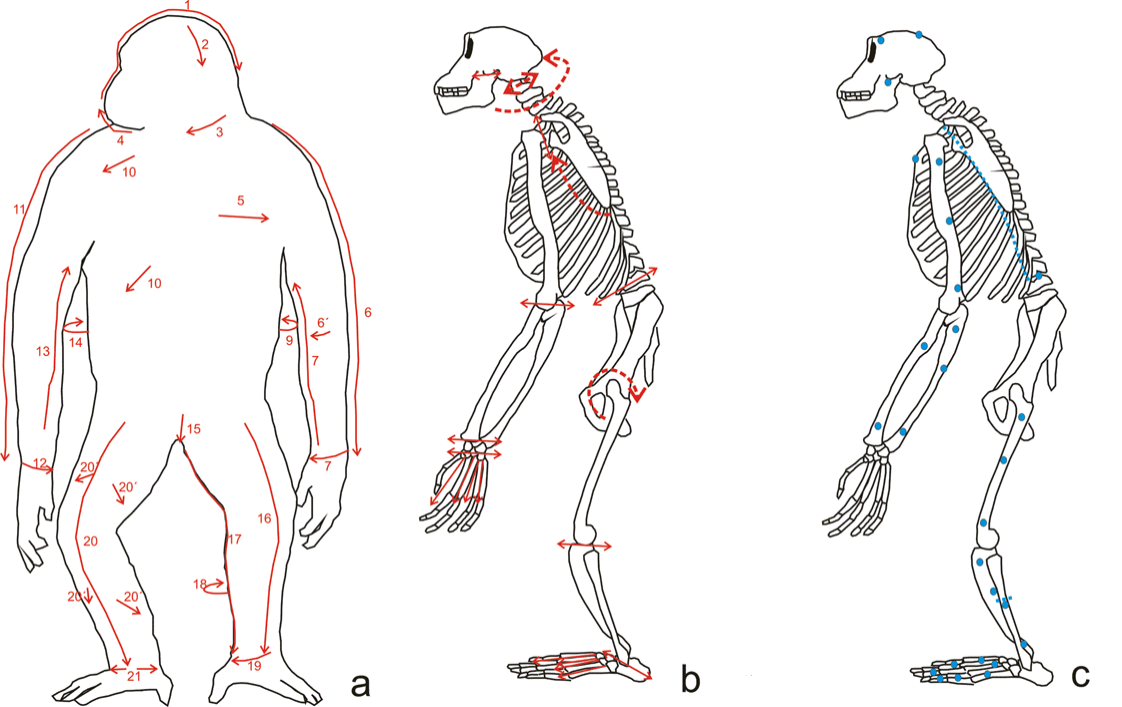
Explanatory scheme of the different steps followed during the butchering process. a) Pattern of the slices and direction of the cuts made during the skinning of the carcass. b) Areas in which the dismembered by segments (dashed line) and the disarticulation of elements (continuous line) was performed. c) Areas where bone was broken by percussion (dots) and where the fracture was performed by bending (dashed line).

The process began with longitudinal slices from the parietals to frontal and orbital area. It then continued with oblique movements from parietal to occipital and, after that, towards the temporal bone in order to remove the right ear. The next step was to extract the lower lip, working below the chin and neck toward the left side of the mandible, removing the upper lip and finishing by extracting the left ear. Removing the skin from the trunk and limbs was easier because it required fewer slices with the tools. Longitudinal cuts were made along the inner side of the limbs, from the chest to the wrist for the arms, and from the abdomen to the ankle for the legs. The process was completed by removing the skin from the back and pelvis (Fig. 2a). During the skinning, lithic tools only came into contact with the skull and jaw bones.

#### Dismembering

The dismembering process consisted of several stages (Fig. 2b). First, the lower limbs were separated from the trunk, then the skull, and finally, the upper limbs were separated from the trunk. The femur and coxa were disarticulated by means of transversal cuts around the femoral head. Cranium and trunk were separated by making transversal cuts at the base of the skull to reach the articulation with the atlas, and around it. Finally, torsion was applied to assist separation. Dismembering of the upper extremities was simple, and only required cutting the flesh around the edge of the scapula.

#### Defleshing

The carcass was defleshed, whenever possible, along the lines of the muscular membranes, separating each of the muscles individually. However, they were sometimes too large or were difficult to differentiate, and in these cases they were cut into portions. The muscles were completely removed from the limbs, trunk and skull, and efforts were made to leave no flesh attached to the bones.

The defleshing of the head began by removing flesh of the face, then of the temporal and occipital bones, and the jaw. The lower extremities were defleshed. Longitudinal cuts on the femurs made it possible to open up the muscle and reach the bone. Transverse and oblique movements were required to remove the muscle. The same process was used on the tibiae and fibulae. Defleshing of the arms began by removing meat from the scapulae with cuts on both the ventral and dorsal faces. On the humeri, longitudinal cuts were made to reach the bone shaft and continued with oblique and transverse cuts. This technique was used on the radii and ulnae, as well. The trunk was defleshed by first removing the flesh from the back and then removing the intercostal meat.

Defleshing involves contact between the tool and the bones, especially in areas where there is less muscle mass, such as the skull. On the long bones, contact usually took place near the epiphysis and midshaft portions of the long bones.

#### Disarticulation

Disarticulation was performed after the main segments had been defleshed, except for the hands and feet. These were processed by first separating the fingers or toes and subsequently defleshing the hand or foot (Fig. 2b). Once this had been done, each metapodial bone was removed from its phalanx, and then each phalanx was separated. The limb bones were disarticulated by making transverse cuts on the bone joints, then applying torsion to the bones whenever possible. The mandible was removed from the skull during the defleshing of the head. Finally, the ribs were separated from the spine by manually bending them and were broken at an angle.

#### Bone breakage

Once the process of soft tissue removal was completed, we proceeded to break the skull, mandible, ribs, long bones, phalanges and metapodials. The technique used for the cranial and appendicular bones was percussion using quartzite hammers and three limestone anvils. One anvil had a flat surface and the other two had angular surfaces; when the flat anvil was used, the bones were placed on the edges. The bones were passive objects in this process, except for the skull for which the passive and active roles were combined; the active movement of the skull proved a more effective breaking technique. The metapodials and phalanges were hit on the shaft to break them and to access the marrow. The ribs were broken by simply bending them, as discussed above (section 3.2). Table 3 contains a summary of the details of the breakage of each bone, and Fig. 2c shows the regions where the bones were percussed or bent.

**Table 3:** Features of the anvil and hammerstone used for the fracture of each bone.

Element	Anvil	Hammerstone	Breakage type	Breakage zone	NISP
**Skull**	Angular anvil of limestone with a flat surface	Quartzite pebble	Support on the flat surface, and the floor. Percussion of the bone against the anvil	Supraorbital torus, zygomatics and parietals	6
**Mandible**	Angular anvil of limestone with a flat surface	Quartzite pebble	Support on the flat surface	Ramus	3
**Humerus right**	Angular anvil of limestone with a flat surface	Quartzite pebble	The bone is supported on one edge of the anvil	Both near epiphyses areas and midshaft	13
**Humerus left**	Angular anvil of limestone with a flat surface	Quartzite pebble	Support on the flat surface	Near distal epiphysis and midshaft	5
**Radius right**	Angular anvil of limestone with central ridges	Quartzite pebble	The bone is supported on one ridge of the anvil	Midshaft and on radial tuberosity	9
**Radius left**	Angular anvil of limestone with central ridges	Quartzite pebble	The bone is supported on one ridge of the anvil	Both near epiphyses areas and midshaft	9
**Ulna right**	Angular anvil of limestone with central ridges and anvil with a flat surface	Quartzite pebble	Combination of support on the angles and the flat surface	Near proximal epiphysis and midshaft	11
**Ulna left**	Angular anvil of limestone with a flat surface	Quartzite pebble	Support on the flat surface	Near proximal epiphysis and midshaft	12
**Femur right**	Angular anvil of limestone with central ridges	Quartzite pebble	The bone is supported on one ridge	Both near epiphyses areas and midshaft	10
**Femur left**	Angular anvil of limestone with central ridges	Quartzite pebble and angular limestone	The bone is supported on one edge	Both near epiphyses areas	8
**Tibia right**	Angular anvil of limestone with central ridges	Quartzite pebble	The bone is supported on one ridge	Near distal epiphysis and midshaft	11
**Tibia left**	Angular anvil of limestone with central ridges	Quartzite pebble	The bone is supported on very sharp edge	Near proximal epiphysis and midshaft	9
**Fibula right**	Angular anvil of limestone with central ridges	Quartzite pebble	The bone is supported on one ridge of the anvil	Midshaft	3
**Fibula left**	-	Quartzite pebble	Flexion	Midshaft	7
**Metatarsals left**	Angular anvil of limestone with a flat surface	Quartzite pebble	Support on the flat surface	Near proximal epiphysis and/or midshaft	20
**Phalanges left**	Angular anvil of limestone with a flat surface	Quartzite pebble	Support on the flat surface	Near proximal epiphysis and/or midshaft	9

The table shows the details of the way of broke, the percussion area and the number of remains of more than 2cm obtained.

## Results

A total of 314 specimens from the chimpanzee skeleton were analyzed (Table 4). Between three and 13 pieces of each long bone were recovered. 52% of the skeletal elements examined exhibited some type of modification caused, 35% with cut marks on their surfaces (Table 5). These marks range in length between 1.2 mm and 50 mm. Cut marks were found alone or in groups of two to 15 striae. The latter were generally parallel or sub-parallel to one another and were oblique and/or transverse in relation to the sagittal axis of the bone.

**Table 4:** Number remains of more than 2cm recovered.

	Right	Left	No position	Total
**Skull**	-	-	6	6
**Mandible**	1	1	1	3
**Vertebrae**	-	-	25	25
**Clavicle**	1	1	-	2
**Sternon**	-	-	3	3
**Rib**	13	35	-	48
**Scapula**	1	1	-	2
**Humerus**	13	5	-	18
**Radius**	10	9	-	19
**Ulna**	10	12	-	22
**Carpal**	10	9	-	19
**Metacarpal**	5	^+^	-	5
**Coxa**	1	1	-	2
**Femur**	9	7	-	16
**Patella**	1	1	-	2
**Tibia**	11	9	1	21
**Fibula**	3	8	-	11
**Tarsal**	5	5	-	10
**Metatarsal**	^+^	20	-	20
**Sesamoide**	4	1	-	5
**Phalange**	35^a^	20^b^	-	55
**Total general**	133	145	36	314

^a^ (phalanges of the hand)

^b^ Phalanges of the Foot.

^+^No processed

**Table 5:** Number of specimens that show cut marks.

	Right	Left	No position	Total	%Total
**Skull**	-	-	5	5	**1.6**
**Mandible**	1	1	-	2	**0.6**
**Vertebrae**	-	-	16	16	**5.1**
**Clavicle**	1	1	-	2	**0.6**
**Sternum**	-	-	-	-	**-**
**Rib**	6	8	-	14	**4.5**
**Scapula**	1	1	-	2	**0.6**
**Humerus**	9	3	-	12	**3.8**
**Radius**	4	2	-	6	**1.9**
**Metacarpal**	2	-	-	2	**0.6**
**Coxa**	1	1	-	2	**0.6**
**Ulna**	7	1	-	8	**2.5**
**Carpal**	1	-	-	1	**0.3**
**Femur**	6	4	-	10	**3.2**
**Patella**	-	-	-	-	**-**
**Tibia**	5	2	-	7	**2.2**
**Fibula**	2	2	-	4	**1.3**
**Tarsal**	1	-	-	1	**0.3**
**Metatarsal**	-	6	-	6	**1.9**
**Sesamoide**	-	-	-	-	**-**
**Phalange**	5	4	-	9	**2.9**
**Total**	**52**	**36**	**21**	**109**	**35.0**

**Fig 3:**
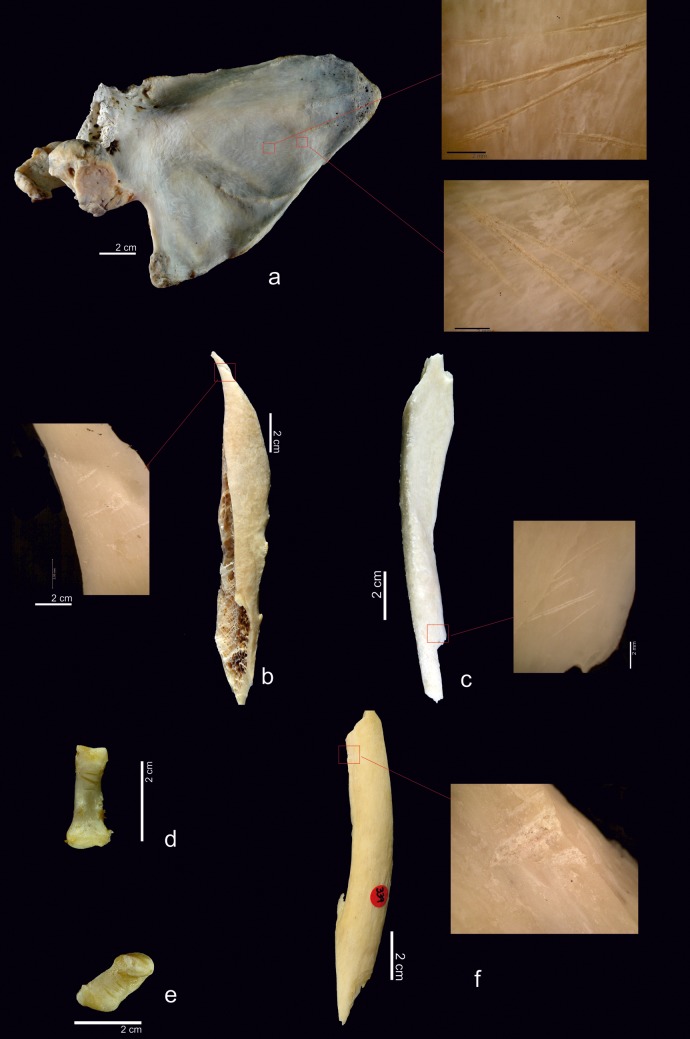
Examples of cut marks on the chimpanzee sample. a) Slicing marks on a scapula. b) Slicing marks on the shaft of a chimpanzee femur. c) Another example of slicing marks on an ulna. d) Phalange with cut marks. e) Cut marks on a pisiform. f) Chop marks on a shaft of femur performed during the fracture of the bone.

Of the cut marks, slicing marks were the most abundant (Fig. 3). These were documented on 106 specimens. Scrape marks were recorded on eight specimens and chop marks on six (Fig. 3). Forty-one of the skeletal elements examined exhibited signatures produced during the hammerstone-anvil bone breakage: 21 percussion pits, 20 percussion notches, and three anvil abrasions. A combination of these modifications was detected on three specimens. Peeling was documented on 37 pieces of the ribs and vertebrae.

### Cranial bones

**Fig 4:**
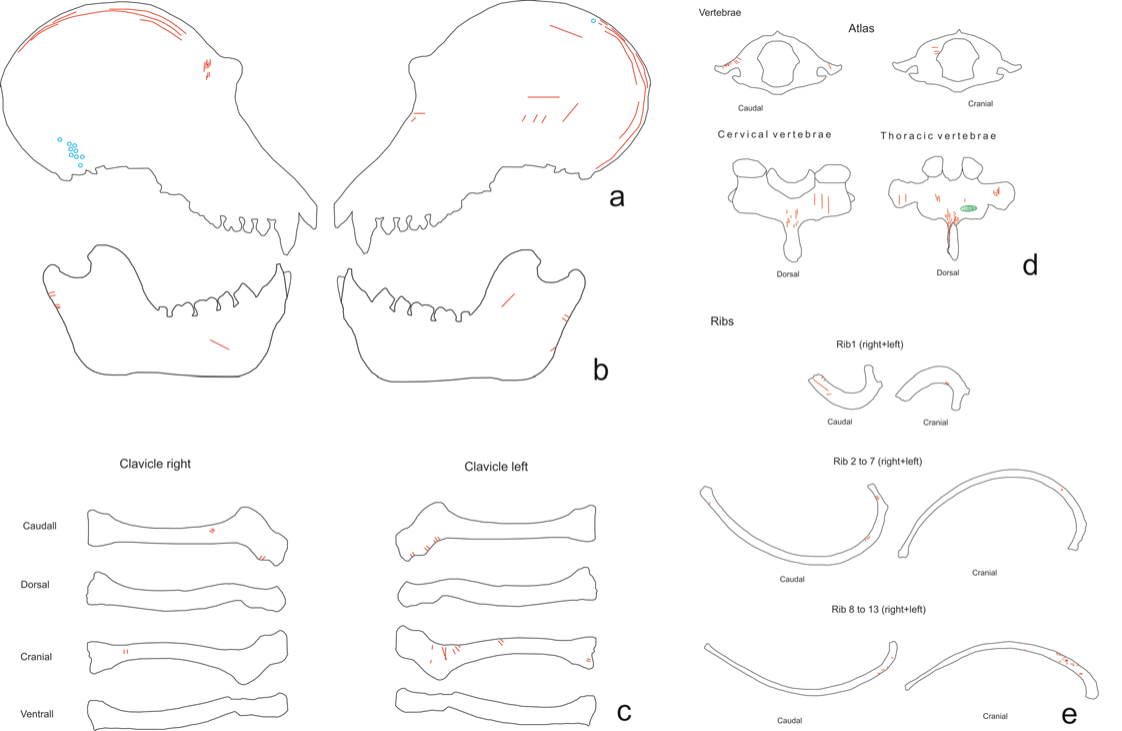
Distribution of the cut marks on: a) skull, b) mandible, c) vertebrae and d) ribs of the chimpanzee sample.

On the skull (NISP = 6, five with cut marks), the marks are related to skinning, defleshing and disarticulation. Most of the slicing marks associated with the skinning process were longitudinal (the longest of these measured 20 mm in length) and were located on the parietal, orbital, temporal and frontal parts of the skull. The sternocleidomastoid muscle was cut in order to remove the ears during skinning. There were some oblique, transversal and longitudinal cuts on the bone which were located on the parietal, frontal and zygomatic bones. These cuts are associated with the removal of the epicranius, temporalis, masseter and orbicularis oculi muscles and caused by the disarticulation and defleshing of the cranium (Fig. 4a).

The mandible (NISP = 3, all with cut marks), exhibited transverse or oblique cut marks. The cuts were located on the vestibular side of the mandibular notch and on the mandibular body (M_1_ zone) and are related to cutting the masseter and depressor anguli oris muscles (Fig. 4b). These marks were therefore identified as caused by the defleshing and disarticulation processes.

**Fig 5:**
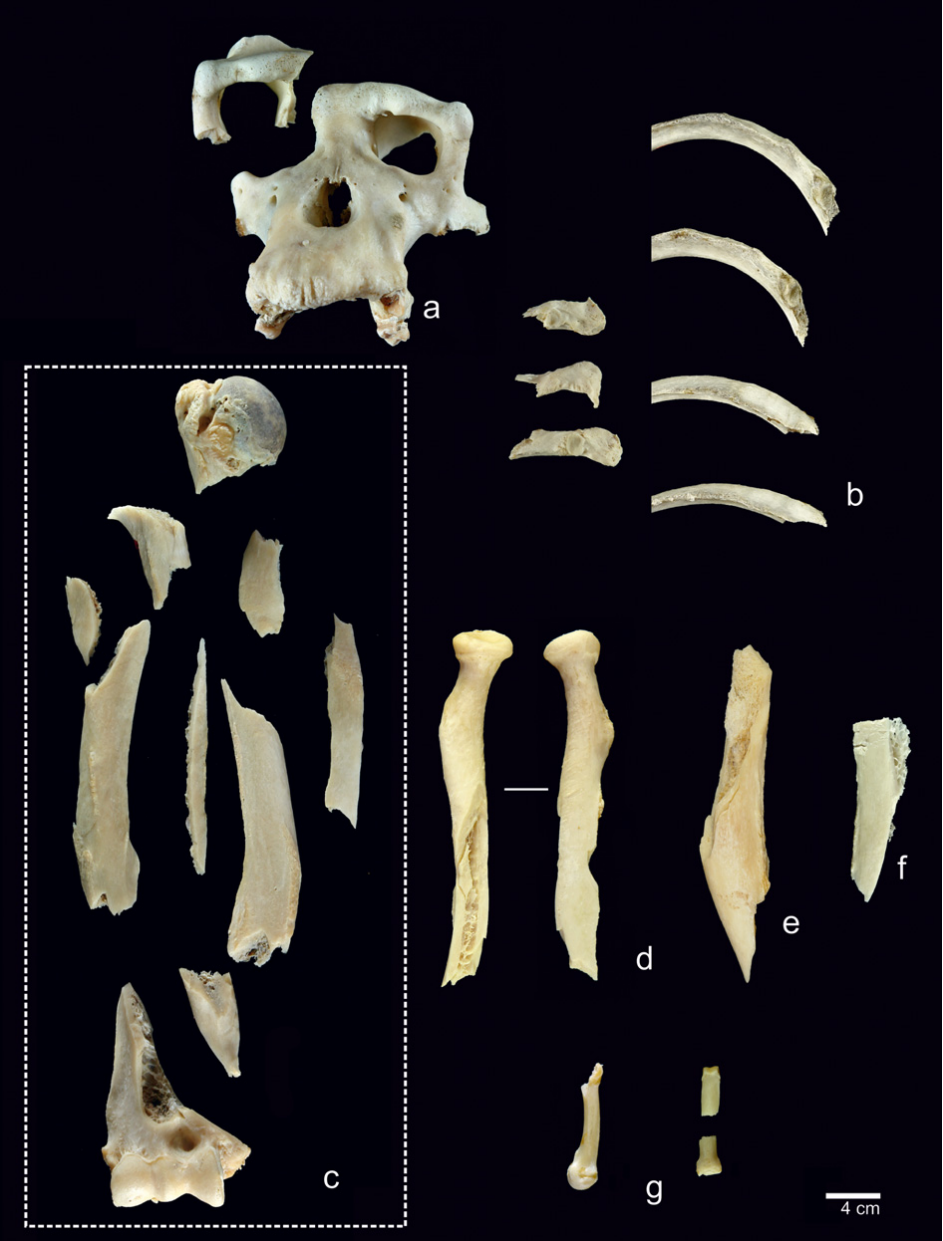
Examples of bone breakage from the chimpanzee sample. a) Two fragments resulting from fracture of the chimpanzee’s face. b) Peeling on the rib angles. c) Fragments resulting from fracturing a humerus. d) Percussion impact on a radius. e) Parasite flake products fracturing a femur. f) Percussion pit on femur fragment.g) Breakage of one metapodial (left) and one phalanx (right).

The calvarium was broken in half lengthwise. Blows to the orbit resulted in the separation of the skull from the face and a right orbital fracture (Fig. 5a). Percussion pits and hammerstone/anvil abrasions were visible on the temporal and parietal bones. Parietal bone shows a cortical scar (23x14 mm) and an adhered flake. Left hemimandible had a transverse fracture on the chin area.

### Trunk

On the vertebrae (NISP = 25, 16 cut with marks), cut marks were documented on cervical and thoracic vertebrae. The atlas exhibited transversal slicing marks on the lamina of the arch and on the transverse process and right pedicle, which were caused by cutting the levator scapulae tensor. These cut marks and the broken left pedicle are due to the disarticulation in which the chimpanzee’s head was separated from the trunk (Fig. 4d). The 5th, 6th and 7th cervical vertebrae exhibited cut marks located on the neural apophyses. These cut marks measured between 1.8 and 3.1 mm and were caused by cutting the multifidus and rotatory muscles during defleshing. All of the thoracic vertebrae, except for the 2nd, had slicing marks in clusters on their neural apophyses (Fig. 4d). The 2nd thoracic vertebra exhibited cut marks on the transverse process, related to cutting the intertransversarii muscle. All cut marks identified on the vertebrae were caused during the defleshing of the back. Peeling was visible on three lumbar vertebrae. One of them showed incipient peeling on the neural apophysis, the other one had general peeling on the articular, transverse and neural apophysis and the third one had general peeling on the neural apophysis.

On the clavicles (NISP = 2, both with cut marks) the cut marks were clustered, and transversal and oblique (Fig. 4c). They were located on the coronoid tubercle, acromial and sternal extremities. Slicing marks were caused during disarticulation, by cutting the pectoralis major, deltoids and sternocleidomastoid muscles.

Cut marks on the ribs (NISP = 48, 14 with cut marks) were mostly clustered and transversal and oblique (Fig. 4e). Scrape marks were visible on one rib. Almost all of the cuts (12) were located on the rib angle area and the diaphyses, but one was located on the neck of the rib. Cut marks documented on these ribs were caused by defleshing and disarticulation. These marks were made during the removal of the external intercostal, serratus anterior and longisimus dorsi muscles. Peeling was found on 32 rib specimens, 14 of them showed general peeling, 12 classical peeling and six incipient peeling. Most of the fractures were at the rib angles (Fig. 5b).

Cuts marks on the coxae (NISP = 2, both with cut marks) were clustered, transversal and oblique. They were located on the lateral and medial sides and on the ischium portion. These marks appeared at the insertions of the quadratus femori and gluteus maximum muscles, indicating that the slicing marks were generated during defleshing.

### Arms and legs

**Fig 6:**
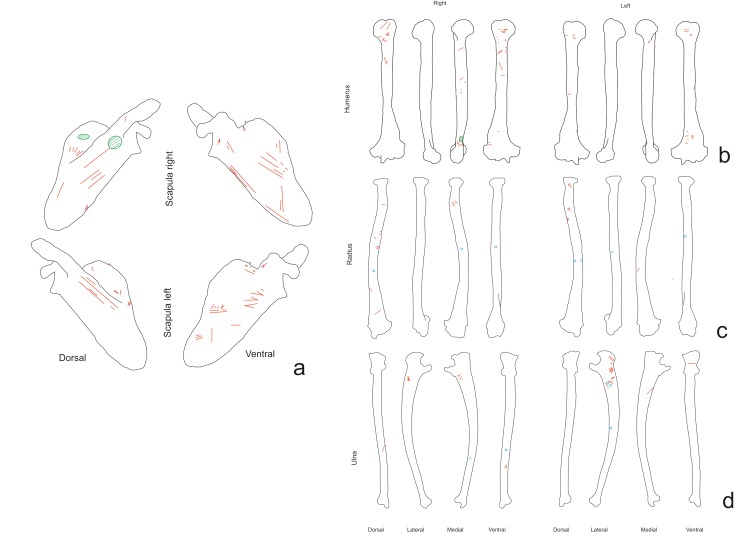
Distribution of the cut marks on fore limb elements. a) Scapulae, b) humeri, c) radii d) and ulna of the chimpanzee sample.

On the scapulae (NISP = 2, both with cut marks), cut marks were mostly clustered (Fig. 6a), and ran in several directions (transversal, oblique and longitudinal) (Fig. 3a). These specimens displayed the longest (50 mm) slicing mark of the sample. The cuts were visible on the dorsal face, on the area where the supraespinatus and infraspinatus muscles attach, and on the scapular spine, where the trapezius, teres major and deltoides muscle attach. The scapular spine had slicing, chop and scrape marks. There were also cut marks on the ventral face and neck of the scapulae. These cuts were caused by cutting the teres minor and subescapularis muscles. All marks identified on scapulae are associated with defleshing and disarticulation.

Cut marks on the humeri (NISP = 18, 12 with cut marks) were clustered, transversal and oblique (Fig. 6b). The slicing marks were along the bone and on the anterior, lateral, medial and posterior sides. The slicing and chop marks were caused by extracting the brachial, the medial and lateral head triceps muscles and the extensor carpo radialis longus. Cut marks were detected on the proximal epiphysis (tuberculus major) caused by cutting the supraspinatus and infraspinatus tendons. Most of cuts were caused during defleshing and the disarticulation of the humeri, the scapulae or the radii ulna.

The radii (n = 19, six with cut marks) exhibited clustered, isolated and located cut marks on the ainterosseous cresta, radial tuberosity and posterior side. Cuts were generated during the removal of the flexor digitorum superficialis and biceps brachii muscles and supinator and pronator tendons, and the slicing marks were caused during defleshing (Fig. 6c).

Cut marks on the ulnae (NISP = 22, eight with cut marks) were clustered and oblique and located on the shaft and all sides of the bones. Most of the cuts were slicing marks, although there were scrape marks on the medial side of the right and left ulnae. Cuts located were made during the removal of flexor digitorum profundus, extensor pollicis longus and flexor carpi ulnaris muscles (Fig. 6d).

**Fig 7:**
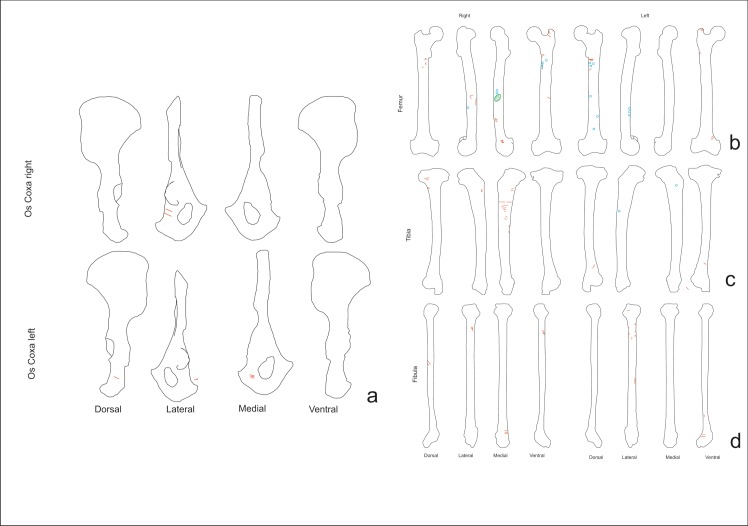
Distribution of the cut marks on the coxa and hind limb elements. a) Coxa, b) femurs, c) tibiae d) and fibulae of the chimpanzee sample.

The femurs (NISP = 18, ten with cut marks) bore clustered, transversal and oblique cut marks located along the length of the bones and on all sides of the bones (Fig. 7b). On proximal epiphyses (trochanter minor and major) there are slicing and chop marks caused by cutting the illiopsoas, gluteus medius, pectioneus tendons and vastus medialis muscles. On the distal epiphyses there were slicing marks related to cutting the gastrocnemius muscle. All these marks on epiphyses were associated with disarticulation. Some of the longer cut marks (+20 mm) were located on shafts and were longitudinal. In addition to these slicing marks, clustered and transverse chop-marks (18 mm) could be observed on the shaft. These slicing marks were caused by cutting the vastus intermedius tendon during defleshing. The chop marks were caused by fracturing the femur (Fig. 5e and 5f).

Cut marks on the tibiae (NISP = 23, seven cut marked) were clustered, transversal and oblique. On the proximal epiphysis and shaft, the cut marks are located where the popliteus tendon, extensor tibial anterior, soleus and plantaris muscles attach. Slicing marks were visible on the distal epiphyses (the fibular notch) and distal shaft; these were caused by cutting the extensor hallucis longus muscle and the Achilles tendon, and are associated with defleshing and disarticulation (Fig. 7c).

The fibulae (n = 11, four cut marked) shows cut marks that were clustered, transversal and oblique (Fig. 7d). On the distal epiphyses and shaft there were slicing marks caused by cutting the peroneus brevismuscle. There were cuts on the proximal epiphyses (crests) caused by cutting the soleus and biceps femoris and peroneus longus muscles. The locations of the cut marks indicate that they were made during disarticulation and defleshing.

All of the long bones were broken (Fig. 5c, d and e), resulting in 107 long bone specimens, of which 41(38.3%) exhibit some type of anthropogenic breakage mark, such as percussion pits, percussion notches, chop marks, adhered flakes and conchoidal scars together with hammerstone/anvil abrasions from the intensive bone breakage process. With one exception, all of the percussion marks were located on the mid shaft portions of the long bones. The exception was a humerus on which a percussion impact was located close to the proximal end.

### Hands and feet

Cut marks were located on six metapodials, two metacarpals and four metatarsals (Fig. 7).

**Fig 8:**
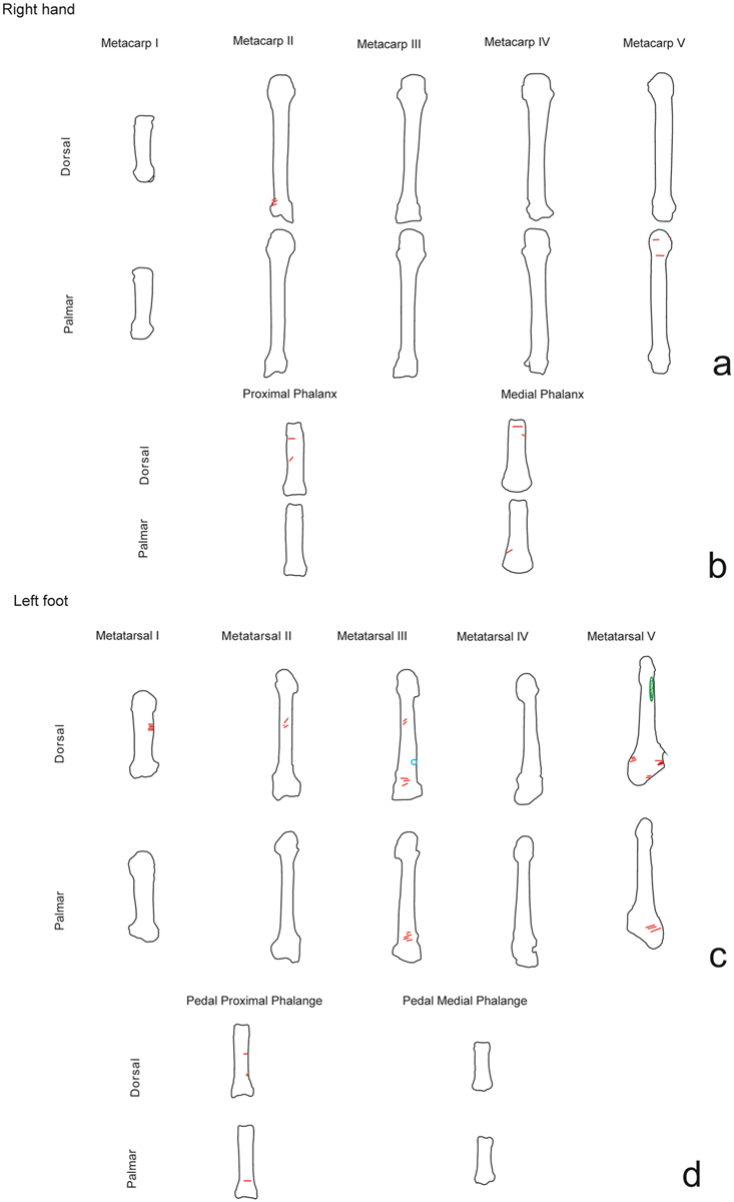
Distribution of the cut marks on metapodials and phalanges of the chimpanzee sample.

On the metacarpals (NISP = 5, two cut marked), clustered and isolated, transversal and oblique cut marks were found on the 2nd and 5th metacarpals (Fig. 8a). On the 2nd metacarpal II, slicing marks were located on the proximal epiphysis and the lateral side. On the 5th metacarpal they were located on the shaft, the distal epiphysis and the palmar side. For both, the cut marks were caused by cutting the flexor digitorum brevis.

On the metatarsals (NISP = 20, four cut marked) the cuts were clustered and oblique (Fig. 8c). They were located along the bone, from the proximal to the distal epiphyses and on the medial, lateral and plantar sides. These marks were caused by cutting the flexor digitorum longus, dorsal interosseus and plantar interosseus muscles. On the plantar side of the 5th metatarsal, a scrape mark was visible that was caused bycutting the plantar interosseus muscle.

On the carpals (n = 19, one cut marked), only the right pisiform bone exhibited transversal slicing marks along the length of the bone and on the anterior and posterior sides (Fig. 3e). These cuts affected the flexor carpi ulnaris, and were therefore caused during disarticulation.

Among the tarsals (NISP = 9, one cut marked), only the chimpanzee’s left calcaneus shows cut marks. The cuts were clustered and transversal. Slicing marks were located on the calcaneal tuberosity and related to cutting the Achilles tendon.

There were nine cut marks on the phalanges (NISP = 72, nine cut marked) (Fig. 3d), five of which were from the right hand and four from the left foot (Fig. 8b and d). On the hand, slicing marks were visible on the 1st, 2nd and 3rd phalanges, located on the medial side and clustered together. These cuts were caused by cutting the flexor digitorum superficialis. On the foot, the cuts were on the 1st and 2nd phalanges. These marks were oblique and are related to cutting the flexor digitorum longus and plantar interosseus muscles. Cuts documented on distal portions of the phalanges were caused during disarticulation and defleshing.

Two phalanges and a 4th metatarsal had percussion pits and hammerstone/anvil abrasions, two of which were impact flakes (Fig. 5f and g). These marks were on the lateral and anterior side shafts. One metatarsal exhibited an adhered flake.

## Discussion and Conclusions

The butchering process is a set of exclusively anthropogenic activities for the purpose of obtaining nutrients and other products derived from animal carcasses. The butchering process begins with accessing the carcass of an animal, and ends with discarding the unused or exhausted remains [53]. The primary goal of the butchering process is to prepare carcasses for transport and consumption, although other ends are also possible, including obtaining secondary materials (e.g. skin or tendons) or extracting and preparing tissues for storage and delayed consumption. The butchering process can be performed on any type of animal, including the corpses of hominins. Before and after the butchering process can have specific aspects associated to the mode of death and how the remains are finally disposed, aspects that can related with ritual treatments of the remains. While it is true that rituals may be present for both animal carcasses and human bodies, it is evident that in this work we are especially interested in the latter. However, whatever the causes and circumstances of the death of the individuals (natural, accidental or violent) and whether or not they were disposed of differently from other animals, the butchering process, whether the end goal is the consumption of humans or other taxa, has the same objectives and would therefore generally be undertaken in the same way. The process includes skinning, defleshing, dismembering, viscera removal, and bone breakage. One of the characteristics that allows us to identify cannibalism is the existence of parallels between the butchering process applied to human bodies and the carcasses of the other animals in an assemblage [3,51,54,55]. In general, most descriptions of European prehistoric cannibalized assemblages describe a butchering process that is similar for humans and animals, as a result of a consistent pattern in the exploitation of meat, bone and brains of the bodies [1,5,8,56–61]. The only possibly ritual processing described in any of these cases was that applied to the skulls in some of the assemblages that contained anatomically modern human remains.

However, many of these assemblages share a common feature in that a high frequency of human remains displays anthropogenic modifications. Between 40 and 60% of the human remains found at El Mirador, Fontbrégoua (France), Gough’s Cave (Great Britain), Moula Guercy (France) and TD6–2 show anthropogenic modifications. Even for the ungulate remains in these assemblages, anthropogenic modifications have been documented on 20% of the specimens [1,4,5,9,55,56]. TD6–2 and MIR 4 assemblages both clearly exhibit this characteristic although there are significant differences between them: the assemblages are from different economic systems (Early Pleistocene hunter-gatherers on the one hand, and a productive Bronze Age society on the other). Additionally, the remains were not disposed of in the same way, the remains at Mirador were in a pit, separate from other taxa and TD6–2 remains were found mixed with the bones of other animals. Lastly, the processing was different, since some were not cooked and others were. Not to mention the almost one million years between the times when the bodies were processed, eaten and disposed of.

These two assemblages do, however, share the high frequency of anthropogenic modifications to the remains recovered, especially cut marks, but also bone breakage. Of the human remains from TD6–2, 44.5% exhibit some anthropogenic modification, whereas 29.7% of the medium size Cervidae remains show signs of a butchering process. In Mirador Cave, 60.1% of the human remains show signals of butchering [1], while only 19.7% were found on the bones of ovicaprini. How these differences in the frequencies of cut marks are interpreted could have significant consequences when attempting to interpret why cannibalism took place in these two assemblages.

In our experiment, the chimpanzee was skinned, defleshed and dismembered, and finally its bones were fractured to simulate a model of making intensive use of all the tissues. The viscera had already been removed, so this part of the process could not be taken into account. The result was that 42.7% of the recovered remains exhibited some kind of human modification. This is close to that observed on the remains found at archaeological sites where cannibalism occurred, and especially close to for the remains from level TD6–2.

In the experimental sample we considered fragments that in an archaeological assemblage could hardly be identified anatomically beyond the more general classifications (long and flat bones) and could not have been assigned to a specific taxonomic group with reasonable certainty. For this reason, we separated out those remains which, in our experience, could not have been identified in an archeological assemblage because of the lack of landmarks (nutrient foramens, muscle attachments, articular facets, twisted parts of bones, etc.). Removing these pieces from the sample changes the percentage of anthropogenically modified bones only slightly (to 44.2%). Therefore, our experimental results do not seem to be affected by the degree to which the remains can be identified.

Any comparison must also take into consideration the fact that the taphonomic history of archaeological remains is more complex, and all of the possible effects of post-depositional modifications cannot be controlled. These include increased fracturing of the remains by diagenesis during the fossilization process. Neither the archaeological assemblage of TD6–2 or the more modern case of Mirador Cave appear to have been significantly affected by taphonomic processes after human activity. In TD6–2, dry fractures affected only 2% of the specimens. The most common post-depositional modification is manganese oxide staining, but this does not affect the bone structure, and so does not affect the degree of anatomical and taxonomic identification or the morphology of the anthropogenic signatures (cut marks or fractures). No signs of carnivore consumption on the remains of *H*. *antecessor* have been documented. In addition, the conservation of some human elements such as vertebrae and ribs, which are likely to disappear in assemblages affected by carnivores, is higher than in the case of other animals, mainly the ungulates [42,62]. This leads us to conclude that the hominin bones were little disturbed after the anthropogenic activity and abandonment.

The post-cannibalistic taphonomic history of the human remains from Mirador Cave has been affected by the activity of the individuals who deposited the bones in the pit, but did not participate in the consumption [1]. However, in this case, the absence of modifications by other agents and/or processes is even more remarkable than in the case of TD6–2. Dry fracturing was only identified on one specimen (0.1%), and post-depositional modifications (trampling, manganese stains and root-etching) affect only 1.3% of the assemblage. As in the previous case, the human remains had not been modified by carnivores [1]. Given all these characteristics, it is appropriate to compare the experimental frequency of the anthropogenic modifications to the chimpanzee’s bones with those documented in these two archaeological assemblages.

Much research in the past ten years has aimed to establish the origin of the different frequencies of cut marks found in archaeological assemblages [21,23–25,27,28], although without much success in most cases. The effects of the raw materials used to make the tools [27] and the number of moves made during the butchering process [21] has been tested, but no clear relationship was established between the frequency of striae and either of these variables. A relationship has been established between the abundance of cut marks and the experience and skill of the butchers [28]. No differences were, however, detected in either the frequency or the locations of the cut marks produced by the two main butchers involved in processing the chimpanzee carcass (ANOVA, F = 0.0614, p =. 806), although it the sample is, admittedly, small.

In the case of archaeological sites, however, it is difficult to assess the number of butchers or their ability. This difficulty is partly due to the fact that the analysis of archaeological assemblages often reveals palimpsests that point to different events occurring during the same or different occupations and involving the same or different individuals, thereby rendering it impossible to objectively evaluate the number of butchers or their experience.

An analysis of the distribution of the modifications to the chimpanzee’s bones revealed that the most of cut marks were located on the muscle attachments and, primarily, on the skeletal segments and elements with the largest muscles or sets of muscles.

**Fig 9:**
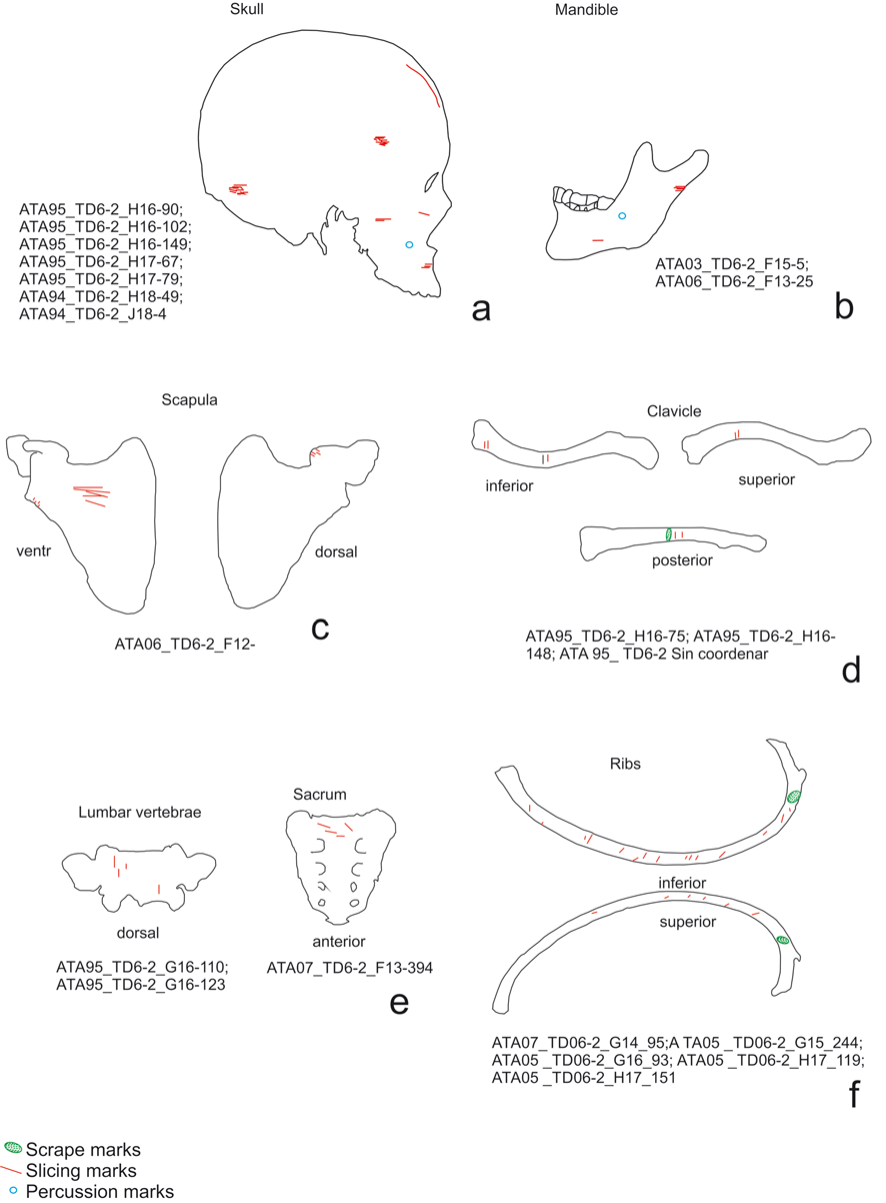
Distribution of cut marks on *Homo antecessor* from TD6–2 elements. a) skulls, b) mandibles, c) clavicles, d) vertebrae, e) ribs.

**Fig 10:**
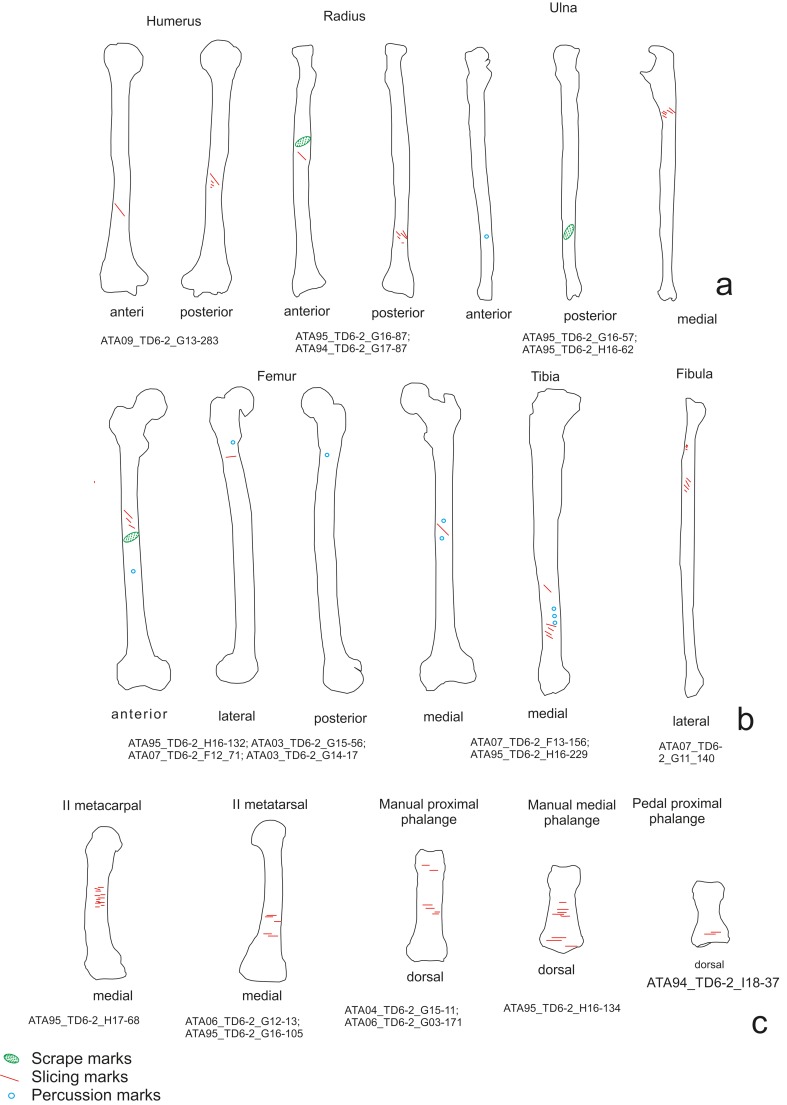
Distribution of cut marks on *Homo antecessor* limb bones. a) humerus, b) radii, c) ulnae, d) femurs e) tibiae, f) fibula, g) metapodials, e) and phalanges.

**Fig 11:**
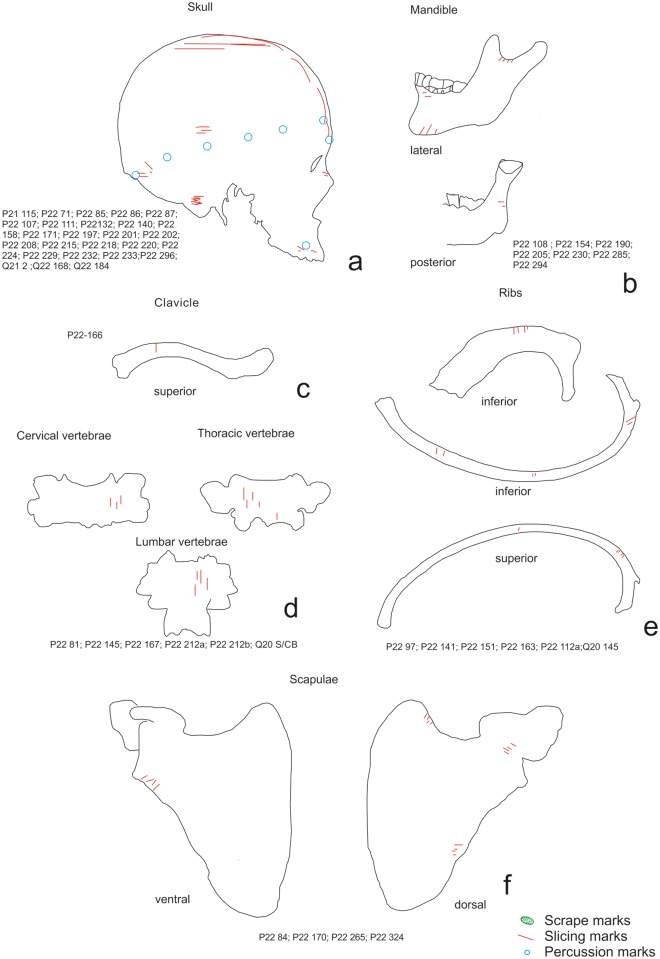
Distribution of cut marks on *Homo sapiens* from MIR4A elements. a) skulls, b) mandibles, c) clavicles, d) vertebrae, e) ribs, f) and scapulae.

**Fig 12:**
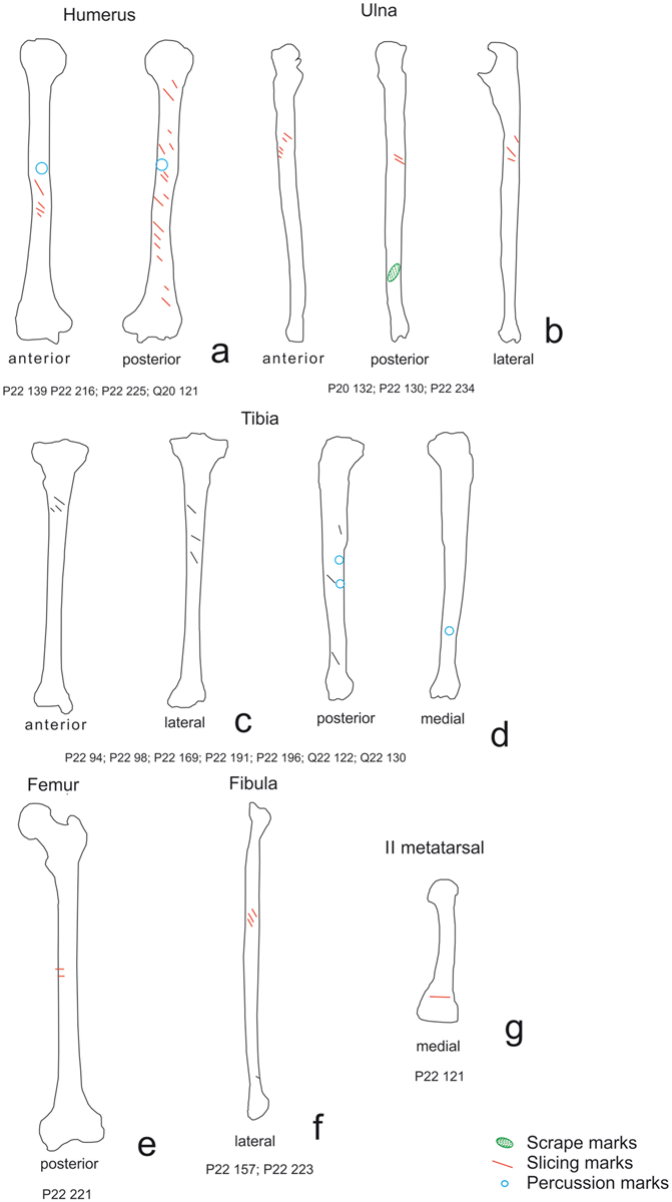
Distribution of cut marks on *Homo sapiens* from MIR4A limb bones. a) humerus, b) ulnae, c) tibiae, d) femurs, e) fibula and f) metatarsal.

The elements with the highest number of marks were the scapulae, humeri, vertebrae and ribs; on the latter there were cut marks associated with removing the flesh from the animal’s back. Regarding the high number of cut marks on the ribs, we must take into account that no marks were caused by evisceration—a process including evisceration would yield an even higher number of marks on the ribs. The remains of *H*. *antecessor* from TD6–2 and *H*. *sapiens* from MIR4 showed cut marks in similar locations [1,4,5] which were mostly caused by removing meat (Figs. 9 to 12). In both cases, the highest number of cut marks on the long bones were located on the shaft; in the case of the ribs, the highest number were located on the proximal part of the shaft; and on the vertebrae the highest number were located on the laminae. In the latter two cases, the cut marks were also due to removal of the meat from these individuals’ backs. Some of the phalanges and metapodials from TD6–2 also exhibit cut marks. This has led to the hypothesis that the tendons in the hands and feet were possibly removed for a specific use [63], although Saladié et al. [5] could not relate these marks to a specific activity. However, the processing of one hand and one foot from the chimpanzee suggests that these cut marks are due to an intensive use of the meat from these extremities.

Skinning has been documented in all three cases on the bones of the skull. However, in this respect there are considerable differences in the calvariae recovered MIR4. The six documented skulls show a pattern of very definite modifications [1]. These modifications were caused by the scalping and fracturing that resulted in what are known as skull cups. This skull breakage pattern has been found in other assemblages associated with ritual cannibalism, as in the case of Gough’s Cave [57], or war cannibalism [59]The remains from TD6–2 do not have these features, and the chimpanzee skull was not processed this way either, even though it was scalped and then fractured in order to extract the brain. This suggests that the calvariae found in Mirador Cave and in other assemblages in which this pattern is present must have been obtained intentionally.

One explanation for the high frequency of cut marks on *H*. *antecessor* and *H*. *sapiens* may be that the bodies were treated differently from how animals were treated. However, the remains of the animals identified in both assemblages were processed in the same way in TD6–2 and MIR-4, except that, at Mirador Cave the human remains were cooked and burned. These similarities extend to our results from processing the chimpanzee. The only notable differences between the human and ungulate samples are related to the skinning of the carcasses. On the ungulates remains, these signs are more common on phalanges or metapodials than on skulls, suggesting that we cannot assume differential treatment. Furthermore, both sets of ungulates underwent the full butchering process, including the removal of the viscera, so these animals’ tissues were also intensively used in both assemblages.

**Fig 13:**
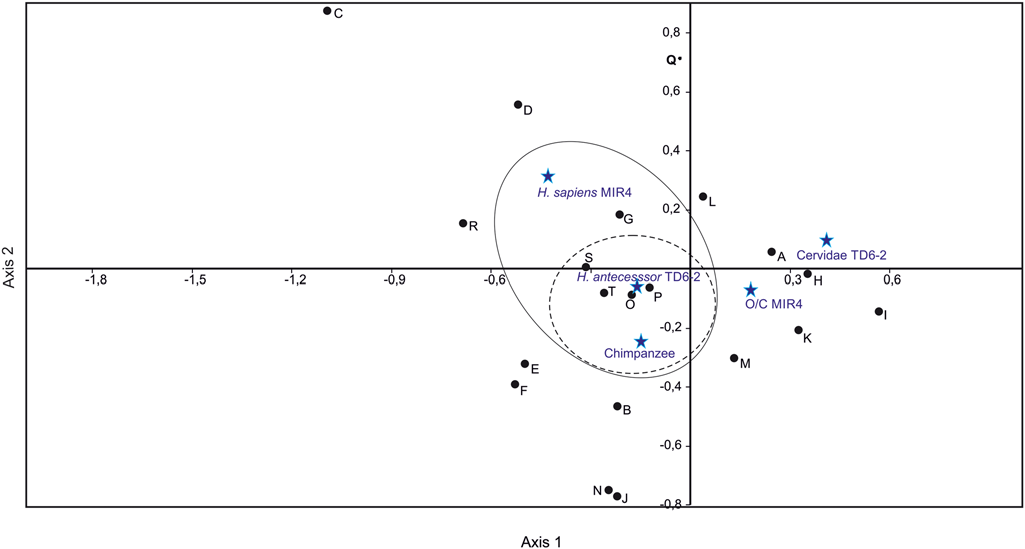
Multiple Correspondence Analysis of cut mark distribution from experimental chimpanzee elements, *Homo antecessor* and Cervidae of TD6–2 assemblage and *Homo sapiens* and ovicaprini of MIR4A assemblages. Figure captions: A = NISP; B = Maxim number of cut marks on one specimen; C = Skull with cut marks (NISP); D = Mandible with cut marks (NISP); E = Ribs with cut marks (NISP); F = Vertebrae with cut marks (NISP); G = Scapulae with cut marks (NISP); H = Humeri with cut marks (NISP); I = Radii with cut marks (NISP); J = Coxa with cut marks (NISP); K = Femurs with cut marks (NISP); L = Tibiae with cut marks (NISP); M = Metapodials with cut marks (NISP); N = Phalanges with cut marks (NISP); O = Remains with defleshing cut marks; P = Remains with disarticulation cut marks; Q = Remains with skinning cut marks; R = Remains with peeling; S = Remains with percussion marks; T = Total of remains with anthropogenic modifications

The question is, therefore, what causes the differences in the frequencies of cut marks? To try to resolve this issue, a correspondence analysis (Fig. 13) was performed to determine whether there was a relationship between the frequency of the cut marks and their anatomical distribution on the chimpanzee specimens, *H*. *antecessor* and the cervids from TD6–2, and *H*. *sapiens* and the ovicaprini from MIR 4. The most discriminating variable is found on Axis 1 (eigenvalue = 59.19), which separates the human and chimpanzee specimens (to the left of the axis) from the ungulates, which are at the right margin of the graph. This separation seems to be related to the higher number of modified axial specimens in the hominin remains. Of the two types of hominin remains studied, a stronger relationship was found between the cut marks on the chimpanzee remains and those on the *H*. *antecessor* remains. The *H*. *sapiens* results are clearly influenced by the number of cranial remains with anthropogenic modifications (Axis 2; eigenvalue = 24.22). Clustering in the cases of the cervids and ovicaprini is marked by the presence of cut marks on limb bones and their reduced presence on the vertebrae and ribs.

This result seems to be related to the frequency of cut marks and the anatomical distribution in the four taxa. Obviously, we had all of the chimpanzee skeletal elements. One of the features that characterize the set of *H*. *antecessor* remains is the presence of ribs, vertebrae and phalanges, defined by Marean and Clerghorn (2003) as low survival elements as they are not typically well preserved in archaeological sites.

The human remains from Mirador Cave mainly consist of calvariae and intensely processed skull fragments. These may have been used for purposes other than providing food, or for purposes in addition to providing food. Processed long bones are present are present in both assemblages. Ungulates from both assemblages are well represented by fragments of shafts of long bones; ribs and vertebrae are also present, but in low numbers, and skull fragments are poorly represented.

These anatomical profiles are also visible in other assemblages in which the frequency of cut marks on the hominin remains is higher than for ungulates. In the sample from Moula Guercy (France), over 50% of the Neanderthal remains exhibit cut marks, while cut marks are found on only 22% of the deer specimens [8,9]. In this assemblage 33.8% of the hominin remains are skull fragments, of which 65.2% (15 of 23) display mark [8] If we also take into account the signals of anthropogenic fracture, 100% of the skulls were modified. In contrast, there is only one modified deer skull fragment in the assemblage.

Another similar case is the Magdalenian assemblage from Brillenhöhle (southwestern Germany) in which 64% of the human remains exhibit cut marks. Orschiedt [10] interpreted this as the result of mortuary practices. This theory is supported by the high frequency of cut marks and their distribution on components of the axial skeleton and on the phalanges. In addition, this assemblage shows obvious parallels with the Magdalenian human remains from Gough’s Cave (Great Britain), where there is clear evidence of cannibalism[56,57]. This anatomical distribution of the cut marks is analogous to that in the TD6–2 assemblage, which shows a high frequency of cut marks on ribs and vertebrae, and these marks have also been documented on phalanges and metapodials [5]. There is no doubt that these modifications are the result of processing and consumption by other hominins and not to subsequent burials.

The case of Mirador Cave is slightly different because of the secondary burial of the human bones, although the modifications are clearly related to processing for consumption, regardless of whether or not there was a ritual treatment. However, the selection of these remains for burial is what made the intensively processed skulls stand out. The frequency of cut marks documented on the chimpanzee bones may be affected by the fact that we have the entire skeleton, including low survival bones, which typically show many cut marks.

The remains from TD6–2 and MIR4 show evidence of specific taphonomic histories that provide insight into the episodes of cannibalism that occurred at the sites, and explain the differences between the skeletal profiles recovered in the two assemblages. At Mirador Cave, most of the human remains were found in a pit in which the remains of other animals were completely absent [1,5]. The remains were clearly intentionally buried, with the skulls carefully placed at the bottom of the pit and the remaining fragments laid on top of them. In this assemblage, there is a marked presence of heavily modified skull specimens.

In TD6–2, the main difference between the *H*. *antecessor* and ungulate samples lies in the fact that while the latter were clearly modified by carnivores, no hominin remains affected by scavenging carnivores have been documented (or the evidence of this is so slight as to be invisible) [42]. This has been interpreted as the result of longer occupations during episodes in which there was cannibalism[42]. The fact that the remains of *H*. *antecessor* were not affected by secondary consumption by carnivores led to the conservation of low survival elements, mainly ribs and vertebrae, the latter of which exhibit numerous cut marks and abundant bone breakage. These data are consistent with the observations made by Domínguez-Rodrigo and Yravedra [29], who stated that the extent of ravaging by carnivores and bone fragmentation are the most influential variables in the total frequency of cut marks.

This study compares the products of three different processes from different contexts (two archaeological and one experimental) and three different skeletal samples (a complete skeleton, one product of different events that did not include ravaging and another that has been biased by the human selection of some bones). However, in all three cases the frequency of anthropogenic modifications is high.

This diversity indicates that it is not only the skeletal profile that determines the frequency of anthropogenic damage, although it definitely plays an important role. The fact that these assemblages have not been ravaged by carnivores is also an important consideration. Furthermore, our results may be affected by the differences between chimpanzee and hominin anatomy. It is clear that the higher or lower frequency of anthropogenic modifications must be judged in each particular context, as suggested by Lyman[26]. There may be different causes that influence the frequency of cut marks: ravaging, type of tools, the size of the carcasses [29] and the skeletal profiles that remain as a result of a variety of causes, but also a possible ritual component. The three cases analyzed here have different taphonomic histories but share the characteristic that they were scarcely disturbed by other agents or processes. Our results indicate that frequencies of anthropogenic modifications after intensive butchering of hominin corpses for food of 30% or higher are not uncommon in different contexts. The experimental butchering process that simulated the cuts made exclusively for the purpose providing food revealed numbers of modifications as high as in archaeological contexts (both where there was ritual behavior and where this was not the case). It can therefore be expected that, in cannibalized and little disturbed assemblages, the frequency of anthropogenic modifications will be high, and will usually affect over 30% of the specimens in the assemblage.
